# Survey of coccidial infection of rabbits in Sichuan Province, Southwest China

**DOI:** 10.1186/s40064-016-2586-6

**Published:** 2016-06-24

**Authors:** Guangwen Yin, Mohsan Ullah Goraya, Juhui Huang, Xun Suo, Zhijian Huang, Xianyong Liu

**Affiliations:** Engineering Laboratory of Animal Pharmaceuticals, College of Animal Science, Fujian Agriculture and Forestry University, Fuzhou, 350002 Fujian Province China; National Animal Protozoa Laboratory, College of Veterinary Medicine, China Agricultural University, Beijing, 100193 China; College of Life Sciences, Fujian Agriculture and Forestry University, Fuzhou, 350002 Fujian Province China

**Keywords:** Coccidial infection, *Eimeria*, Rabbits, Southwest China

## Abstract

Coccidiosis is a challenging disease of wild and domestic rabbits both, caused by Eimeria and thereby leads enormous economic losses at rabbit farms. The present study carried out to survey the prevalence and intensity of coccidial infection among the rabbits in Sichuan Province, southwest China. A total of 110 faecal samples were collected from 11 farms situated in eight main rabbits rearing administrative regions. Oocysts in faecal samples were purified, sporulated and identified according to morphological features. The overall prevalence of infection was 56.4 % (62/110), with prevalence of 64 % (47/75) for local meat breeds of rabbit and 51.4 % (18/35) for Rex Rabbits (local fur rabbits). Weanling rabbits had the highest prevalence (74 %, 37/50), followed by young rabbits (45 %, 13/29) and the adult rabbits showed the lowest prevalence (42 %, 13/31). Concurrent infection with two to seven Eimeria species was found. In total, 9 species of Eimeria were identified from oocyst-positive samples. *E. perforans* was the most prevalent specie (42.73 %), followed in order by *Eimiera media*, *E. irresidua, E. magna*, and *E. intestinalis* with prevalences of 35.45, 34.55, 31.82, and 23.64 %, respectively. Results of the present investigation indicated that the prevalence of coccidial infection is high among the rabbit population in southwest China. This study also elucidate about the coccidial infection and emphasis to adopt control strategies in commercial rabbitories.

## Background

Coccidiosis is a pervasive parasitic disease that can infect number of animal species. Animals which could be infected by the coccidians include chickens, dogs, camel, rabbit, cats, cattle and sheep. However, different species of Eimeria causing coccidiosis infect different animals and different organs. Coccidiosis is one of the major parasitic diseases in commercial rabbit production. Wild rabbits are more prone to infection as compared to the local meat rabbits. There are two types of rabbit coccidiosis intestinal and hepatic (Coudert 1989). Though, more than twenty-five species of Eimeria have been reported that could infect the rabbit (Bhat et al. 2010). However, until now 17 species of them has been identified which infect rabbit (*Oryctolagus cuniculus*) (Duszynski and Couch 2013). Though, it is well established that about 10 species colonized in the intestinal tract and one species (*Eimeria stiedai*) infecting the biliary ducts of the liver (Coudert 1989; Kvicerova et al. 2008; Oliveira et al. 2011). The disease directly influences the production potential of infected rabbits due to high mortality, retarded growth and poor feed conversion ratio, leading to high economic losses to the industry every year (Varga 1982; Pakandl et al. 2008a). Additionally, in sub-clinical form, it may render the rabbits immune-compromised that paves the way to secondary disease conditions. All domesticated rabbit breeds can be infected by coccidia, especially the younger populations between 1 and 4 months of age (Pakandl et al. 2008a; Pakandl 2009). Coccidians can invade and destroy intestinal cells of the hosts, causing anaemia, electrolyte imbalance and poor absorption of nutrients (Szkucik et al. 2014; Metwaly et al. 2013).

China is the largest rabbit-producing country in the world, it produced approximately 700,000 tons of rabbit meat in 2009, which constituted more than 40 % of the world yield in that year (Dalle Zotte and Szendro 2011). The Sichuan Province is the major rabbit producing area, holding the first position with maximum number of rabbits and highest meat production in the country. It produces about 40 % of the rabbit meat in China. Some surveys have shown that *Eimeria* infection in rabbits is common in some provinces of China (Qiao et al. 2012; Pan et al. 2008). So far, there is no reported data regarding prevalence of rabbit coccidial infection in Sichuan Province, which has unique climatic and geographic conditions different from other provinces.

Hence, the objective of the study is to investigate the prevalence of coccidial infection at various stages of life and by different species of Eimeria. The survey was conducted based on the data from local meat and Rex Rabbit breeds in Sichuan Province. Findings of this study can facilitate the understanding of disease occurrence and to design efficient control system for rabbit coccidiosis in the area.

## Methods

### Selection of rabbitories

The study was conducted among the rabbit populations in Sichuan Province, southwest China, which is located between the northern latitudes of 26°03′ to 34°20′ and eastern longitudes of 97°22′ to 110°10′. This province has an average annual temperature of 16 °C in the east and 8 °C in the west, and the average annual rainfall ranges from 1000 to 1300 mm.

Fecal samples were collected randomly from 11 different farms of 8 divergent regions in the province (Table 1) and examined for the presence of oocysts. In these farms, faecal samples were collected from 75 different groups of local meat rabbits and 35 of Rex Rabbits. While to consider the age factor 50 samples were collected from weanling rabbits (1–3 months old), 29 from growing rabbits (3–6 months old) and 31 samples were from breeding rabbits (older than 6 months).

**Table 1:** Prevalence and intensity of coccidia infection in different regions of Sichuan Province

Regions	Examined no.	Positive no.	Prevalence (%)	OPG
Jiangyou	10	9	90.0	17,800
Meishan	10	3	30.0	13,400
Chengdu	40	25	62.5	21,100
Mianzhu	10	1	10.0	4800
Deyang	10	4	40.0	14,400
Leshan	10	5	50.0	17,000
Luzhou	10	7	70.0	63,400
Bazhong	10	8	80.0	40,400

### Sampling and treatment method

A total of 110 faecal samples were collected randomly (random numbers table method) from apparently healthy animals of 8 main rabbits rearing administrative regions. From each chosen population, 500 g of fresh faecal pellets were collected as one sample. All samples were stored at 4 °C and transported to the laboratory (National Animal Protozoa Laboratory, College of Veterinary Medicine, China Agricultural University, Beijing, China) for further analysis. Each faecal sample was homogenised in 500 ml tap water, and then 2 g of the mixture was put into 60 ml of saturated salt solution (Mundt et al. 2005; Velkers et al. 2010). The suspension was then emptied into a modified McMaster chamber to check the oocysts, and the oocyst per gram (OPG) was calculated to estimate the degree of infection (Coudert and Drouet-Viard 1995). The limitation of detection value was set as 200 oocysts per gram faecal sample.

### Species identification

Oocysts from each faecal sample were purified as previously described (Coudert and Drouet-Viard 1995; Kvicerova et al. 2008) and sporulated by placing on a shaker and diluted into a 2.5 % potassium dichromate solution at 28 °C for 7 days to ensure good aeration. Concentrated oocysts in each sample were identified based on their sizes and morphological characteristics (shape, colour, form index, presence or absence of the micropyle and its cap, presence or absence of residual, polar and Stieda bodies) of the oocysts and sporocysts (Kvicerova et al. 2008; Coudert and Drouet-Viard 1995). To ensure that species identification is valid, at least 50 sporulated oocysts from each species were observed and measured.

### Statistical analysis

The statistical package SPSS was used for data analyses, and a value of P < 0.05 was considered significant difference in comparison.

## Results

### Prevalence of coccidial infection in rabbits of Sichuan

A total of 110 samples were collected and analysed. Overall, coccidian oocysts of Eimeria were found in 62 of 110 faecal samples (56.4 %) obtained from the eight regions of Sichuan Province. The prevalence of coccidian oocysts in the eight regions ranged from 10.0 to 90 % (Table 1). Jiangyou region had the highest prevalence (90 %) and Mianzhu region had the lowest prevalence (10 %). The morphological identification of *Eimeria* oocysts revealed the presence of nine species of *Eimeria*, namely*, E. stiedai, E. magna, Eimeria irresidua, Eimeria media, Eimeria piriformis, Eimeria intestinalis, Eimeria flavescens, Eimeria coecicola,* and *Eimeria perforans.E. perforans* was the most prevalent species (42.7 %), followed in order by *E. media*, *E. irresidua, E. magna*, and *E. intestinalis* with prevalences of 35.45, 34.55, 31.82, and 23.64 %, respectively (Table 2).

**Table 2:** Percentage of faecal samples infected with each *Eimeria* species

Species	Examined no.	Positive no.	Prevalence (%)	OPG
*E. magna*	110	35	31.8	13,471
*E. media*	110	39	35.4	7913
*E. coecicola*	110	9	8.18	4055
*E. intestinalis*	110	26	23.6	9530
*E. perforans*	110	47	42.7	8221
*E. irresidua*	110	38	34.6	11,168
*E. piriformis*	110	7	6.36	3571
*E. flavescens*	110	7	6.36	14,014
*E. stiedai*	110	1	0.91	1200

### Phenotypic of Rabbits and prevalence of Eimeria

Coccidian oocysts were found in 64 % (47/75) of faecal samples from local meat rabbits and 51.4 % (18/35) from Rex rabbits, these values were not significantly different (P > 0.05) (Table 3). Local meat rabbits had the higher OPG value (27,820) than the Rex rabbits (13,540) (P > 0.05).

**Table 3:** Prevalence and intensity of coccidia infection in different rabbit breeds in Sichuan Province

Breed	Examined no.	Positive no.	Prevalence (%)	OPG
Meat rabbits	75	47	64.0	27,820
Rex rabbits	35	18	51.4	13,540

The prevalence of coccidian oocysts in weanling rabbits (74 %) was higher than in the growing rabbits (45 %) and breeding rabbits (42 %). All the differences were statistically significant (P < 0.05) (Table 4). The intensity of infection in weanling rabbits (38,480) was also significantly higher (P < 0.05) than that in young rabbits (10,400) and breeding rabbits (9540), which is consistent with previous observations (Pakandl et al. 2008b).

**Table 4:** Prevalence and intensity of coccidia infection in adult and young animals in Sichuan Province

Age groups	Examined no.	Positive no.	Prevalence (%)	OPG
Breeding rabbits	31	13	42.0	9540
Growing rabbits	29	13	45.0	10,400
Weanling rabbits	50	37	51.5	38,480

### Concurrent infections of Eimeria

**Fig. 1:**
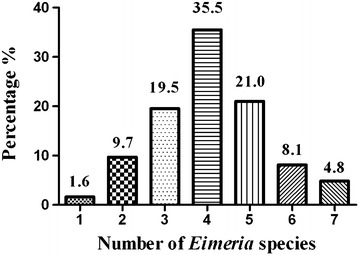
Percentages of single and mixed infections with different *Eimeria* species

The percentages of single and mixed infections of different *Eimeria* species in rabbits are shown in Fig. 1. Concurrent infections with more than one *Eimeria* species commonly presented in the current rabbit populations. Most of the rabbits carried two to seven species. *E. perforans, E. media* and *E. magna* were the species most frequently found in concurrent infections.

## Discussion

In the present study, the prevalence of coccidia infection in eight regions of Sichuan Province was surveyed. Based on the analysis of 110 faecal samples collected from 11 rabbit farms, the overall infection rate was 56.4 %. Due to high pressure of disease there is continuous use of coccidiostats at farm level. In spite of the use of coccidostats prevalence of the disease is still high. The existence of 9 *Eimeria* species was confirmed in these faecal samples. The prevalence was higher than that at the country-wide level in China (Jing et al. 2012), which revealed a prevalence of 41.9 % in rabbits. This difference may come from due to several reasons. In Sichuan Province, grass, silage and grain are more widely used as rabbit feed in many small farms, making it difficult for small holders to administer coccidiostat as compared to commercial farmers. In Sichuan province, numbers of small farm holders are more as compared to rest of China. In small farms, poor hygienic condition and suboptimal temperatures are also favorable for *Eimeria* infections (Schlolaut et al. 2013; Jing et al. 2012) which put rabbit population at more risk to coccidiosis in Sichuan Province.

*E. stiedai, E. magna, E. irresidua, E. flavescens, E. piriformis* and *E. intestinalis* are regarded as more pathogenic coccidia in rabbits (Polozowski 1993; Coudert and Drouet-Viard 1995). Although *E. magna, E. irresidua* and *E. intestinalis* were the dominant species in the examined faecal samples, most of the OPG values for these samples were less than those corresponding to clinical coccidiosis. This result indicates that sub-clinical coccidiosis is common in Sichuan Province. Sub-clinically infected rabbits look to be healthy in general, but may have less feed consumption, reduced feed conversion and growth performance, resulting in huge economic losses for rabbit production industry. A further detailed survey is extremely needed to unleash the contribution of coccidia infection to economic losses in future.

The prevalence of coccidian oocysts in local meat and Rex rabbits was not significantly different (P > 0.05), which suggests that the breed may have little influence on the prevalence of coccidiosis in rabbit production. The prevalence of coccidian oocysts in weanling rabbits (74 %) was higher than that in growing rabbits (45 %) and breeding rabbits (42 %), and the intensity of infection in young animals (weanling rabbits) was significantly higher (P < 0.05) than in adults (breeding rabbits), This could be due to lower resistance or less immunity to coccidian oocysts in young rabbits compared to elder animals as described previously (Pakandl et al. 2008a, b).

## Conclusions

In conclusion, the present survey revealed that prevalence of rabbit coccidial infection in Sichuan Province is high. Knowledge of the prevalence of coccidiosis and current Eimeria species will help to evaluate the infection potential and control programs, and thus to minimize the economic losses in the rabbit production industry. These results also provide relevant “base-line” data for assessing the effectiveness of future control strategies against rabbit coccidiosis.
